# Short-Term Betanin Intake Reduces Oxidative Stress in Wistar Rats

**DOI:** 10.3390/nu11091978

**Published:** 2019-08-22

**Authors:** Davi Vieira Teixeira da Silva, Aline D’Avila Pereira, Gilson Teles Boaventura, Roberto Stefan de Almeida Ribeiro, Maurício Afonso Verícimo, Carla Eponina de Carvalho-Pinto, Diego dos Santos Baião, Eduardo Mere Del Aguila, Vania M. Flosi Paschoalin

**Affiliations:** 1 Instituto de Química, Universidade Federal do Rio de Janeiro, Avenida Athos da Silveira Ramos 149, Rio de Janeiro 21941-909, RJ, Brazil; 2 Departamento de Nutrição e Dietética, Universidade Federal Fluminense, Niterói 24020-140, Brazil; 3 Departamento de Imunobiologia, Universidade Federal Fluminense, Niterói 24020-150, Brazil

**Keywords:** beetroot, hyperlipidemia, lipid peroxidation, oxidative stress, antioxidant activity, hepatic damage reversal

## Abstract

Oxidative stress is a common condition described in risk factors for cardiovascular disease. Betanin, a bioactive pigment from red beetroot demonstrates anti-inflammatory and antioxidant properties. The main aim of this study was to evaluate the short-term intake of betanin against oxidative stress in a rodent model, a common condition described in several risk factors for cardiovascular disease. Oxidative stress was induced in Wistar rats by a hyperlipidemic diet for 60 days, followed by betanin administration (20 mg·kg^−1^) through oral gavage for 20 days. Plasma biochemical parameters and antioxidant enzyme activities were evaluated. Lipid peroxidation and histopathological changes were determined in the liver. The hyperlipidemic diet caused hyperglycemia, hyperinsulinemia, insulin resistance, and increases in alanine transaminase and aspartate transaminase levels. Oxidative stress status was confirmed by reduction of antioxidant enzyme activities, increased lipid peroxidation, and liver damage. Purified betanin regulated glucose levels, insulin, and insulin resistance. Hepatic damage was reversed as evidenced by the reduction in alanine transaminase and aspartate transaminase levels and confirmed by histological analyses. Betanin reduced hepatic malondialdehyde and increased superoxide dismutase, catalase, and glutathione peroxidase activities. Short-term betanin intake modulated biochemical parameters, reversed hepatic tissue damage, and attenuated oxidative stress in Wistar rats.

## 1. Introduction

Cardiovascular diseases (CVD) are the worldwide leading cause of death. They comprise a class of disorders that involve blocking blood supply to cardiac muscle and the brain due to the presence of inflammatory cells, such as macrophages and T lymphocytes, as well as deposition of oxidized lipids in the vascular wall of blood vessels [1,2]. Disorders such as coronary heart and cerebrovascular diseases are the major clinical manifestations of CVD [3].

Several pathologies, such as risk factors for CVD, including hypertension, hyperlipidemia, and diabetes, have in common a disruption of the redox status. Excessive production of reactive oxygen species (ROS) occurs in all vascular diseases [4,5,6,7]. Although ROS are constantly generated in the mitochondrial respiratory chain and play an important role as regulatory mediators in signaling processes, cell proliferation and defense, and gene expression, their excess, caused by an imbalance between their production and cell detoxification capacity, establishes a pathophysiological condition termed oxidative stress [8].

It is recognized that oxidative stress both promotes and is induced by vascular diseases, via a retro-feeding mechanism. When oxidative stress is stimulated in the blood vessels, it is followed by adverse vessel reactivity, comprising vascular smooth muscle cell proliferation, macrophage adhesion, platelet activation, and lipid peroxidation [9,10,11].

Reactive oxygen species (ROS) are chemical species containing oxygen that are formed under physiological conditions due to the partial reduction of molecular oxygen. Under physiological conditions, ROS are removed by the defense mechanisms exerted by antioxidant enzymes, the main ones being intracellular superoxide dismutase (SOD), which converts O_2_^−^ to O_2_ and H_2_O_2_ anions, and catalase (CAT) and glutathione peroxidase (GPx), which convert H_2_O_2_ to water (H_2_O) and O_2_ [12,13,14]. However, when produced in excess, ROS can cause damage to lipid structures, low-density lipoprotein (LDL)-cholesterol oxidation, induction of inflammatory and immune responses, vascular tone alteration, protein and DNA oxidation, metabolic dysfunctions, and cell signaling interruption [6,15,16,17].

In addition to risk factors for CVD, substantial evidence suggests that diets rich in carbohydrates and excessive fat consumption increase ROS generation, promoting oxidative stress [18,19]. Hyperlipidemia may lead to mitochondrial dysfunction, increased ROS production, as mentioned previously, and reduced expression of antioxidant enzymes [20,21,22]. This indicates that oxidative stress plays an important role in CVD progression and should, therefore, be the target of strategies aiming at the prevention and treatment of these disorders.

On the other hand, epidemiological evidence and clinical trials demonstrate that, in addition to the endogenous antioxidant defense system, vegetable consumption displays a protective effect on oxidative stress and CVD, due to the bioactive antioxidant phytochemicals that make up the nutrients of these food matrices [23,24,25,26]. Antioxidants may exert their effect on biological systems via different mechanisms, including electron donation, metal ion chelation, co-antioxidants, or gene expression regulation [27]. Betalains are a class of compounds that contain a betalamic acid in their general chemical structure, accompanied by a radical R1 or R2, where the substituents may be a simple hydrogen or a more complex radical [28,29].

Betanin is a heterocyclic compound most abundant within betacyanins found in beetroot (*Beta vulgaris* L.), conferring its red-violet coloring [30,31]. The antioxidant power of betanin (betanidin 5-*O*-β-d-glucoside), the major betalain, lies in the presence of cyclic amine groups and a hydroxyl (–OH), which are good hydrogen and electron donors, with the capacity to stabilize reactive species. The antioxidant activity of betalains can be increased according to the number and position of the hydroxyl groups in the molecule, with the C-5 position of the hydroxyl group in the aglycone responsible for increasing their antioxidant activity [29,32]. In addition to its effect in reducing ROS formation, betanin emerged as a phytochemical able to reduce the expression of inflammatory cytokines and genes encoding oxidant enzymes, as demonstrated in cultured human cell lines and in rodent models, where betanin was able to modulate metabolic disorders and degenerative modifications in different organs and tissues [33,34].

In fresh beetroot juice, betanin is found at a concentration of 1.19 g·mL^−1^ [31]. The chemical stability and antioxidant ability of this compound are preserved during simulated human gastrointestinal digestion. Half of ingested betanin molecules can reach the small intestine digestive fluid and may be absorbed at the mucosa border cells. The high antioxidant ability inherent to betanin was retained even after a simulated small intestine digestion. Betanin displayed peroxyl-radical scavenger ability and could prevent lipid oxidation in pork meat when assayed in vitro [31].

However, the in vivo effects previously attributed to betanin from red beetroot were claimed using the whole red beetroot extract, commercially available [35,36]. The red beet extract contains several phytochemicals and bioactive compounds found in the vegetal matrix, including polyphenols and organic acids, in addition to betanin. For this reason, the beneficial effects of beetroot extract cannot be attributed uniquely to betanin.

The present study had the purpose of testing the effect of short-term purified betanin supplementation in Wistar rats with oxidative stress fed a hyperlipidemic diet. Furthermore, the effects of betanin intake on carbohydrate and lipid metabolism, antioxidant enzyme activity, and lipid peroxidation, as well as the histopathological changes in hepatic tissue induced by oxidative stress, were also evaluated.

## 2. Materials and Methods

### 2.1. Reagents

Insulin ELISA kit, 1,1,3,3-tetramethoxypropane ((CH_3_O)_2_CHCH_2_CH(OCH_3_)_2_) 2-thiobarbituric acid (C_4_H_4_N_2_O_2_S), butylated hydroxytoluene (BHT), 2-thiobarbituric acid (C_4_H_4_N_2_O_2_S), and diethyl pyrocarbonate (O(COOC_2_H_5_)_2_) were purchased from Sigma (Sigma-Aldrich Co, St. Louis, MO, USA). Blood biochemical analytical kits (total cholesterol, triglycerides, glucose, aspartate aminotransferase, and alanine aminotransferase) were purchased from Bioclin (BioclinQuibasa, Belo Horizonte, MG, Brazil). GPx, CAT, and SOD assay kits were purchased from Cayman Chemical (Cayman Chemical Company, Ann Arbor, MI, USA). Betanin was purified from fresh beetroot juice [31].

### 2.2. Feed Stuff Processing

The AIN-93G and AIN-93M feed offered to the animals was produced according to the American Institute of Nutrition for Rodent diets [37]. A modified AIN-93G and AIN-93M feed with 60% of calories originated from lipids was prepared by mixing starch, soy oil, lard, sugar, and l-cysteine. The ingredients were purchased at the local Rio de Janeiro municipality trade, in southeastern Brazil. Casein, a vitamin mix, minerals, colin, and cellulose were purchased from PragSoluçõesBiociências (SP, BRA). Standard feed comprised 18% kcal as fat, 20% kcal as protein, and 62% kcal as carbohydrate, with a caloric value of 349 kcal·100 g^−1^. High-fat feed comprised 60% kcal as fat, 14% kcal as protein, and 24% kcal as carbohydrate, with a caloric value of 474 kcal·100 g^−1^. Table 1 presents the composition and concentration of ingredients in both the standard and high-fat diets.

### 2.3. Animals

The experimental animal protocol was approved by the Federal Fluminense University (UFF) Ethics Committee on the Use of Animals, Niterói, Brazil, under no. 773/2016. The animal procedures are in accordance to the Brazilian School of Animal Experimentation—National Council for Animal Experiments Control (CONCEA). Weaned 21-day-old male Wistar rats (70 ± 2.3 g) were obtained from the Laboratory Animal Core-UFF.

**Figure 1:**
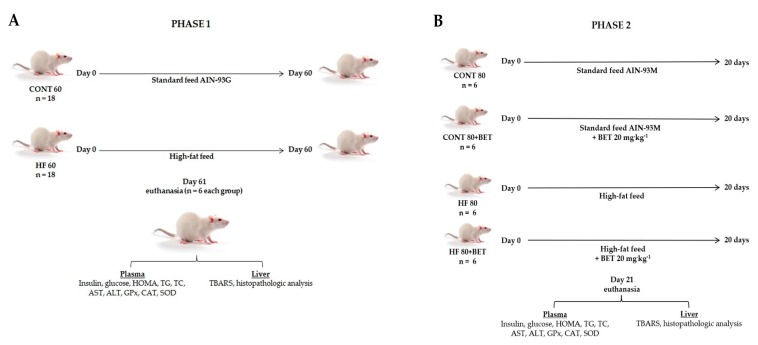
Experimental design of the study. (**A**) phase 1: CONT 60 - control group 60 days of standard AIN-93G feed; HF 60 - high-fat group 60 days of high-fat feed. (**B**) phase 2: CONT 80 - control group 80 days of standard AIN-93M feed; CONT 80+BET - control group 80 days of standard AIN-93M feed + betanin by 20 days; HF 80 - high-fat group 80 days of high-fat feed; HF 80+BET - high-fat group 80 days of high-fat feed + betanin by 20 days.Glu: glucose; HOMA: homeostatic model assessment; TG: triglycerides; TC: total cholesterol; AST: aspartate aminotransferase; ALT: alanine aminotransferase; GPx: glutatione peroxidase; CAT: catalase; SOD: superoxide dismutase; TBARS: thiobarbituric acid reactive substances.

The study was carried out in two phases, where all animals were fed ad libitum and maintained in collective cages under controlled temperature (21–23 °C) and a light–dark cycle (12/12 h). In phase 1, 36 rats were divided into two groups: the control group (CONT 60, *n* = 18), fed the standard feed for 60 days, and the high-fat group (HF 60, *n* = 18), fed the hyperlipidemic feed during the same period (Figure 1A).

At the end of the 60 days, the establishment of the oxidative stress condition was evaluated. Animals (*n* = 6 each group) were fasted (12 h), before being anesthetized with isoflurane, and blood samples were collected by cardiac puncture for glucose, insulin, triglyceride (TG), total cholesterol (TC), aspartate transaminase (AST), alanine transaminase (ALT), GPx (glutathione peroxidase), catalase (CAT), and SOD (superoxide dismutase) determinations. Livers were fractionated and frozen (−80 °C) for thiobarbituric acid reactive substance (TBARS) determination, and one fragment of each lobe was fixed in 10% buffered formalin, embedded in paraffin, cut into 5-µm slices using an automated RM2265 microtome (Leica, DEU), and stained with hematoxylin–eosin (H&E) for histopathological analyses.

Phase 2 of the study lasted an additional 20 days to assess the effect of betanin intake in animals fed the standard AIN-93M or hyperlipidemicdiets. The remaining 12 animals in the control group were divided into two groups (*n* = 6), where six animals were fed the standard feed for another 20 days (CONT 80) and the other six were fed the standard feed plus betanin (CONT 80+BET). Similarly, for the group of animals fed the high-fat feed, six animals were fed the high-fat feed for an additional 20 days (HF 80) and the other six were fed the high-fat feed plus betanin (HF 80+BET). Betanin was administered by intra-gastric gavage at a concentration of 20 mg·kg^−1^, because it was the dose capable of regulating liver enzymes in diabetic rats [36], and the control group received water. After 20 days of treatment, all animals were fasted (12 h) and submitted to the same procedures as the phase 1 study (Figure 1B).

### 2.4. Plasma Analyses

#### 2.4.1. Biochemical Analyses

Blood samples were collected in citrate tubes and centrifuged at 3000× *g* for 15 min to separate the plasma. Insulin was determined using an enzyme-linked immunosorbent (ELISA) assay kit. Glucose, triglycerides (TG), total cholesterol (TC), aspartate transaminase (AST), and alanine transaminase (ALT) were determined using biochemical analysis kits according to the manufacturers’ instructions. Insulin resistance was determined as described previously by a homeostasis model assessment (HOMA-IR), using the following equation: insulin (µU·ml^−1^) × glucose (mmol·L^−1^)/22.5 [38].

#### 2.4.2. Antioxidant Enzyme Activities

GPx, CAT, and SOD activities were determined by colorimetric methods on a Victor X4 spectrophotometer (Perkin Elmer^®^, Waltham, MA, USA) using commercially available kits (Cayman Chemical Co). GPx activity was determined at 340 nm by measuring the decrease in reduced nicotinamide adenine dinucleotide phosphate (NADPH) absorbance using hydrogen peroxide as substrate [39,40]. CAT activity was determined at 540 nm using methanol hydrogen peroxide as substrate [40]. SOD activity was determined at 450 nm using xanthine oxidase and hypoxanthine as substrates, according to Peskin et al. [41]. GPx, CAT, and SOD enzyme activities were expressed as U·mL^−1^.

### 2.5. Tissue Analyses

#### Thiobarbituric Acid Reactive Substances (TBARS)

Lipid peroxidation was evaluated by a TBARS assay as an indicative of malondialdehyde (MDA) concentration and oxidant damage [42]. Liver samples (3.0 g) were washed with 0.9% NaCl, and MDA was extracted using 9 mL of 7.5% trichloroacetic acid (TCA) and 50 µL of 7.2% butylated hydroxytoluene (BHT). The homogenates were centrifuged at 3000× *g* for 15 min and filtered through Whatman grade 4 filter papers (Merck-Millipore Co). The filtrates were used as the MDA extract. Subsequently, 1 mL of sample was derivatized with a 20 mM thiobarbituric acid (TBA) solution, and the absorbance of the MDA–TBA adducts was determined at 532 nm on a DU^®^530 spectrophotometer (Beckman Coulter Inc., Brea, CA, USA). The results were expressed as µM MDA·g^−1^, using 1,1,3,3-tetramethoxypropane (TMP) asan MDA standard.

### 2.6. Histopathological Analyses

Histopathological alterations were evaluated by comparing morphological changes in liver tissue between healthy animals and oxidative stress-induced animals, treated or not with betanin. Liver H&E-stained (hematoxylin and eosin) sections were examined at 20× and 40× magnifications under an optical microscope.

### 2.7. Statistical Analysis

Assessment of significant differences concerning plasma insulin, glucose, HOMA, TG, CT, enzymatic activities (AST, ALT, GPx, CAT, and SOD), lipid peroxidation (TBARS), and histopathological alterations between the CONT 60 and HF 60 groups during phase 1 of the study was performed by an unpaired *t*-test. One-way analysis of variance (ANOVA) with repeated measurements was performed to identify the differences in each biochemical parameter in plasma and hepatic tissue of the CONT 80, CONT 80+BET, HF 80, and HF 80+BET groups during phase 2 of the study. Differences were set at a confidence level of 0.05, and additional post hoc tests with Bonferroni adjustments were performed. Values were expressed as means ± standard deviation (SD), and statistical analyses were carried out using the Prisma software version 5 for windows (GraphPad Software, San Diego, CA, USA).

## 3. Results

### 3.1. Biochemical Parameter Analyses

Glucose, HOMA-IR, TC, TG, and AST increased after phase 1 in the animal group fed the HF60 when compared to the group fed the CONT 60 (Table 2). In phase 2 of the study (Table 3), animals were fed their respective feed for an additional 20 days (CONT 80 and HF 80) or were treated with 20 mg·kg^−1^ betanin (CONT 80+BETand HF 80+BET). The HF 80 group displayed increased plasmatic glucose, insulin, HOMA-IR, TG, AST, and ALT when compared to the other groups. However, no difference in TC was observed between the HF 80 and HF 80+BET groups. Furthermore, glucose, insulin, HOMA-IR, TG, AST, and ALT were reduced in HF 80+BET animals when compared to the HF 80 group. In addition, co-ingestion of betanin promoted improvement in ALT values in both CONT 80+BETand HF 80+BET.

### 3.2. Antioxidant Enzyme Activities

**Figure 2:**
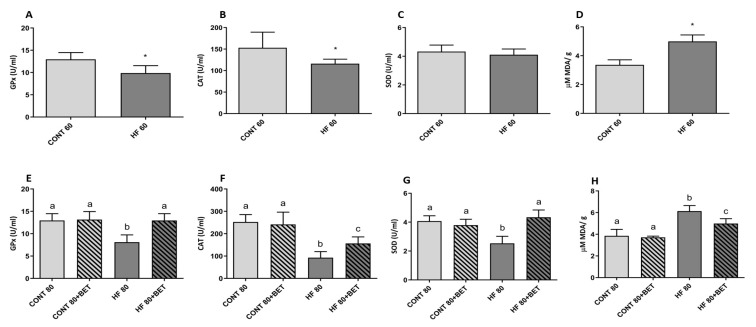
Antioxidant activity of glutathione peroxidase (GPx) (**A**), catalase (CAT) (**B**), superoxide dismutase (SOD) (**C**), and liver malondialdehyde (MDA) (**D**) concentrations after phase 1 and GPx (**E**), CAT (**F**), SOD (**G**), and MDA (**H**) after phase 2 of high-fat feed intake. The symbol* in Figure 2A,B,D indicates differences between the CONT 60 (control group fed the standard feed AIN-93G)and HF 60 (high-fat group fed the hyperlipidemicfeed)at a *p* < 0.05 significance level. Different letters in Figure 2E–H indicate differences at a *p* < 0.05 significance level.

The high-fat feed ingestion led to decreased enzymatic GPx (CONT 60: 12.9 ± 0.6 vs. HF 60: 9.86 ± 0.7 U·mL^−1^) and CAT (CONT 60: 153.1 ± 14.8 vs. HF 60: 116 ± 4.3 U·mL^−1^) activities when compared to the control group (Figure 2A,B). However, no change was observed in SOD activity (CONT 60: 4.32 ± 0.2 vs. HF 60: 4.10 ± 0.2 U·mL^−1^) (Figure 2C). After phase 2, no changes in GPx, CAT, or SOD were observed when compared to animals fed the control feed supplemented with betanin (CONT 80 and CONT 80+BET) (Figure 2E–G). However, the HF 80+BET group displayed increased GPx activity when compared to the HF 80 group (12.9 ± 1.54 vs. 8.11 ± 1.6 U·mL^−1^, respectively) (Figure 2E). Betanin supplementation in the HF 80+BET group also increased CAT and SOD activities when compared to the HF 80 group (156.3 ± 29.6 vs. 92.7 ± 27.6 and 4.52 ± 0.2 vs. 2.86 ± 0.7 U·mL^−1^, respectively) (Figure 2F,G). Despite the increase in CAT activity in the HF 80+BET group in comparison to HF 80, CAT activity was maintained at lower levels compared to animals fed the control feed (Figure 2F).

### 3.3. Thiobarbituric Acid Reactive Substances (TBARS) in Liver Tissue

MDA concentrations increased in animals belonging to the HF 60 group when compared to the CONT 60 group (4.99 ± 0.18 vs. 3.36 ± 0.14 µM, respectively) (Figure 2D). After phase 2, an increase in MDA levels was observed in the HF 80 group compared to the CONT 80, CONT 80+BET, and HF 80+BET groups. Similarly, betanin co-ingestion inhibited MDA increases in animals fed the high-fat feed compared to the HF 80+BET and HF 80 groups (5.13 ± 0.14 vs. 6.21 ± 0.54 µM, respectively) (Figure 2H).

### 3.4. Histopathological Analyses

**Figure 3:**
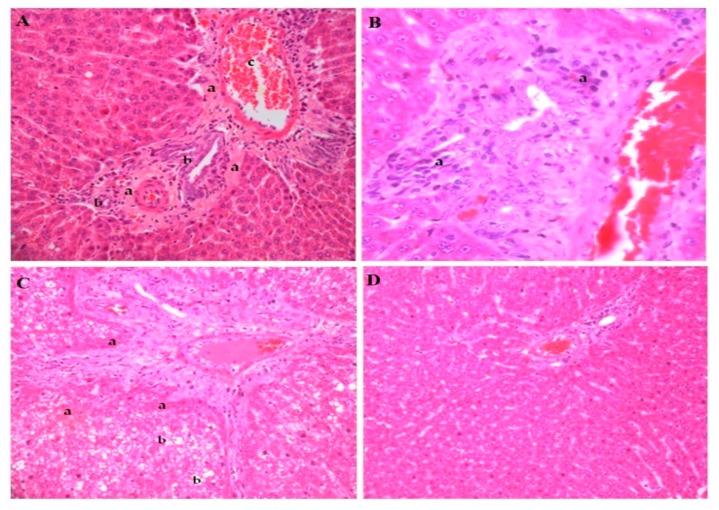
Histopathological liver alterations of rats fed the high-fat feed during phase 1 (**A**–**C**). Panel **A** shows the thickening of connective tissue capsule in the portal triad (a), proliferation of bile ducts (b), anddilation of the portal vein branch (c). Panel **B** indicates the presence of mononuclear cell inflammatory infiltrates (a); hematoxylin and eosin(H&E), 40×. Panel **C** indicates the presence of hepatocyte necrosis areas (a) and micro and macro vesicular steatosis (b). Panel **D** represents the liver of animals fed the standard feed AIN-93G. Photographs were recorded at 20× magnification (H&E staining).

The histological images of the hepatic tissue of healthy animals fed the standard feed (CONT 60) were compared to animals fed in phase 1 (HF 60) and phase 2 (HF 80), and those fed with hyperlipidemic feed supplemented with betanin (HF 80+BET). The animals from the HF 60 group presented connective tissue thickening in the portal triad, bile duct proliferation, portal vein dilation, presence of mononuclear cell inflammatory infiltrates, and extensive hepatocyte areas of steatosis and necrosis (Figure 3A–D).

**Figure 4:**
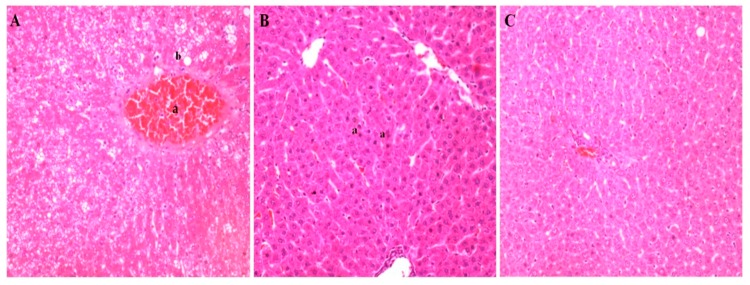
Histopathological liver alterations of rats fed the high-fat feed during phase 2 (Panel **A**) and the high-fat feed plus betanin (Panel **B**). Panel **A** displays centrilobular vein congestion (a), macro vesicular degenerations, andnecrosis (b). Panel **B** indicates normal hepatocytes suggestive of a regenerative cellular process in the high-fat group supplemented with betanin (a), with a histological architecture resembling healthy animals from the control group (Panel **C**). Photographs were recorded at 20× magnification (H&E staining).

After phase 2, in addition to the morphological alterations, centrilobular vein congestion and necrosis were also observed in the animals in the HF 80 group, indicating damage in the venous return (Figure 4A). Betanin co-ingestion reduced lipid accumulation in hepatocytes and reversed the changes promoted by high-fat feed ingestion. The liver displayed preserved histological structures, such as the portal triad, hepatocytes, and central lobular vein, compatible with those from a healthy animal, including hepatocytes suggestive of a regenerative process (Figure 4B,C).

## 4. Discussion

The high-fat diet offered for 60 days led to changes in glucose metabolism, an increase in insulin resistance confirmed by HOMA-IR, increased cholesterol and triglyceride plasma levels, and liver damage confirmed by histopathological analyses.

The established hyperglycemia can promote mitochondrial fragmentation and reduce the activity of the electron transport chain, consequently increasing ROS production [43]. In addition, hyperglycemia and insulin resistance promote oxidative stress via the mitochondrial respiratory chain as the primary source of the superoxide anion (O_2_^•−^). The mitochondrial O_2_^•−^ mediates the protein kinase C stimulus and the generation of advanced glycation end products (AGEs), a complication of diabetes [44]. Protein kinase C and AGEs in turn can activate NADPH oxidase and inhibit endothelial nitric oxide synthase (eNOS). In addition, eNOS uncoupled via NADPH oxidase promotes extra oxidative stress due to the higher production of O_2_^•−^ [45,46].

Elevated cholesterol and triglyceride levels, together or alone, can accelerate the development of atherosclerosis and are associated with high lipid peroxidation as a consequence of high ROS production [7,47,48].

Considering the existence of evidence that an imbalance in the redox status was established, caused by changes in glucose and lipid metabolism, it can be concluded that administration of the high-fat feed by 60 days caused oxidative stress in the evaluated animals.Biomarkers such as GPx, CAT, and SOD are used as indicators of oxidative stress (disruption of the redox status) [4]. This disruption in the redox state was confirmed by the reduction of the activity of antioxidant enzymes, such as GPx and CAT. Furthermore, there was an increased lipid peroxidation in the liver in response to steatosis and excess substrate (lipids) for peroxidation, and tissue oxidative damage was evidenced by histopathological alterations in the liver and increases in plasmatic liver health-related biomarkers, AST and ALT. The results described herein corroborate previous studies such as those carried out by Sarna et al. [49], González-Mañán et al. [50], and Venezuela et al. [22], who indicated impaired glucose metabolism, increased hepatic liver injury enzyme markers, lipid peroxidation, and reduction in antioxidant enzyme activities in high-fat fed animals.

Inflammation is also appreciated for its role in many diseases, including atherosclerosis [1]. Currently, significant interest is noted in identifying novel therapeutic strategies to target oxidative stress considered as a critical, final common mechanism in the physiopathological conditions that contribute to risk factors for CVD. Beetroot and its formulations are acclaimed for their established beneficial effects on the vascular endothelium via the nitrate (NO_3_^−^)–nitrite (NO_2_^−^)–nitric oxide (NO) pathway [30]. However, beetroots are the main source of betanin, and this major red-violet beetroot pigment emerged as a powerful antioxidant phytochemical [28]. Studies in animal modelswere more conclusive about the plasma bioavailability and biological effect of purified betanin intake [35,51]. Betanin and betalain bioavailability in humans is considered low; however, the betanin mechanism regarding absorption, metabolism, and excretion routes, still requires elucidation. Silva et al. [31] evaluated betanin bioavailability during in vitro human simulated gastrointestinal digestion via continuous multistage steps. The authors observed an important decrease in betanin content after the gastric simulated digestion, reaching 65% of the initial sample content, and lowering to 46% after small intestine simulated digestion. Furthermore, no betanin was detected after the ex vivo colon fermentation assay, where the remaining betanin recovered at the end of the in vitro simulated gastrointestinal digestion, corresponding to 54% of the original sample, was assayed by ex vivo colon fermentation. This 35% decrease in betanin content observed after gastric digestion is due to its impaired stability at acidic pH 2. It is known that betalains exhibit stability at pH ranging from 3–7. In acid pH, the betanin structure can be degraded via C-17 decarboxylation, dehydrogenation, and cleavage of betalamic acid into cyclo-dopa-5-*O*-glycoside [31].

The administration of betanin for 20 days was able to regulate glucose and insulin levels and reverse insulin resistance, even under the concomitant deleterious stimulus caused by the high-fat feed. The results described herein reinforce the powerful effect of betanin on the carbohydrate metabolism in diabetes, as reported previously, as the administration of betanin administered at 25 and 100 mg·kg^−1^ for 60 days was able to revert hyperglycemia, hyperinsulinemia, HOMA-IR, and glycation products in animals with experimental diabetes induced by high fructose ingestion [35]. Similar positive effects were described for the glucose metabolism in rats displaying streptozotocin–nicotinamide-induced diabetes, where treatment with betanin at 10, 20, and 40 mg·kg^−1^ for 30 days was evaluated, and the betanin at 20 mg·kg^−1^ (the same dose used in the present study) was the most effective in altering glucose and insulin levels, by regulating key glucose metabolism enzymes in liver, such as glucokinase, pyruvate kinase, glucose-6-phosphate dehydrogenase, glucose-6-phosphatase, and fructose-1,6-bisphosphatase, when compared with betanin at 10 and 40 mg·kg^−1^ [36].It is important to note that, in the present study, the biological efficacy of betanin during short supplementation was assessed, while most studies evaluated its effect in vivo for periods of 30 days or longer. Herein, 20 days of betanin intake promoted beneficial outcomes similar to 30- and 60-day treatments as reported by Han et al. [35] and Dhananjayan et al. [36].

Betanin was not able to reduce plasma cholesterol levels but prevented the increase of triglycerides in the HF 80+BET group compared to the HF 80 group, maintaining TG levels close to those of healthy animals. To the best of our knowledge, the study conducted by Wroblewska et al. [52] is the only one to demonstrate the hypolipidemic effect of betalain on cholesterol and triglycerides, but the effect was promoted via whole-beet crisps, which contain betacyanins and betaxanthins, making it unfeasible to attribute the effect to a single compound. Thus, betanin probably has no proven lipid-lowering effect; however, its effect on triglyceride reduction observed herein may be due to decreased hyperglycemia and, consequently, lower triglyceride synthesis in response to glucose metabolism regulation [53].

Elevated levels of serum AST and ALT enzymes are indicative of liver damage, where ALT is considered a better liver damage marker, as it is predominantly found in hepatocytes [54,55]. The high-fat feed provoked a deleterious effect on the hepatocytes of animals fed during phases 1 and 2, as confirmed by the aforementioned increases in ALT and AST, as well as histopathological changes. On the other hand, betanin administration for 20 days reduced plasma AST and ALT levels and restored carbohydrate and lipid metabolism, as well as the characteristic architecture of liver cells even under the stimulation of a hyperlipidemic diet.

Betanin was demonstrated as having a close relationship with hepatic tissue protection, since the healthy animals of the control group supplemented with betanin displayed a decrease in plasmatic ALT levels, a good hepatocyte integrity marker, in relation to healthy animals not supplemented with betanin. Betanin supplementation to high-fat diet-fed animals also led to decreased ALT levels, which were lower than those fedstandard feed and much lower than in HF animals untreated with betanin. These results corroborate previous in vivo reports which indicate that betanin exerts a hepatoprotective effect, modulating cytochrome P450 CYP2E1, AST, and ALT and reducing mitochondrial damage [56].

Several oxygenated chemical compounds, particularly MDA and conjugated dienes, are produced during free-radical attacks on membrane lipoproteins and polyunsaturated fatty acids, when GPx, CAT, and SOD enzymes display reduced activity [57,58]. Pathological conditions that predispose cardiovascular events, such as hypertension, hypercholesterolemia, and diabetes, are associated with upregulation and downregulation of oxidant and antioxidant enzyme messenger RNA (mRNA), being the probable mechanism for the GPx, CAT, and SOD decreased activities found in the present study. However, betanin intake at 20 mg·kg^−1^ positively and significantly influenced the oxidant status of Wistar rats, reducing hepatic MDA concentrations and restoring antioxidant enzyme activities. Betanin was demonstrated to influence antioxidant enzyme activity, non-enzymatic protein and lipid peroxidation glycation, and inflammatory and fibrosis statusreduction in rat heart and kidney [59,60]. The antioxidant effect exerted by betanin and its positive promotion of a redox balance lies not only in its ability for electron and hydrogen donation, but also in mRNA expression modulation. Betanin can reduce the expression of CYP3A2 and the inflammatory cytokines inducible nitric oxide synthase (iNOS) and cyclooxygenase-2 (COX-2) in animals exposed to paraquat [60,61,62]; it is also able to inhibit gene expression in rodent and human cell lines. Betanin also inhibited the expression of NADPH oxidase 4 (NOX-4), an important endogenous ROS generator, in tubular renal cell cultures, and was reported to regulate the expression of transcription factor erythroid 2-related factor 2 (Nrf2) and phase II enzymes involved in ROS detoxification and elimination, including glutathione *S*-transferase A (GSTA), GSTP, GSTM, GSTT, NAD(P)H:quinone oxidoreductase 1 (NQO1), and heme oxygenase-1 (HO-1) [34,60,61,62]. This evidence may support a possible role of betanin as an activator of the GPx, CAT, and SOD genes, enhancing the amount of available enzyme molecules to exert their antioxidant activities, as evidenced in the present study.

Herein, the animals fed a hyperlipidemic diet presented disorders in glucose and lipid metabolisms, in addition to liver injury, increased lipid peroxidation, and reduced antioxidant enzyme activities, clinical conditions that alone or in combination lead to oxidative stress similar to that observed in patients with CVD risk factors, favoring the development and progression of the disease. Betanin intake during a short period was able to ameliorate the metabolic disturbances that lead to oxidative stress and tissue injury. It is important to emphasize that an increase in consumption of fruits and vegetables is recommended as a key component of a healthy diet for the prevention of chronic diseases. The dose–response relationship between fruit and vegetable consumption, cardiovascular risk, and cancer mortality was quantified in a recent meta-analysis, providing further evidence that the higher consumption of fruit and vegetables, and consequently antioxidants, is associated with a lower risk of all-cause mortality, mainly cancer and cardiovascular diseases [23]. In addition, previous chronic human studies using high amounts of beetroot and, consequently, high doses of betanin, NO_3_^−^, phenolic compounds, and organic acids, through juice, gel, and cereal bar intake provided biochemical benefits and vascular effects without adverse outcomes [63,64,65]. Furthermore, to reach the beneficial biochemical effects, betanin, for the intake of other bioactive compounds, requires a supplementation period to reduce oxidative stress parameters [35,36]. Han et al., [35] observed that 100 mg·kg^−1^ intake of betanin for 60 days antagonized the changes of plasma glucose, insulin, HOMA, and glycated hemoglobin marker levels, demonstrating the antifibrotic role of betanin against fructose-induced diabetic cardiac fibrosis in Sprague–Dawley rats. Betanin also decreased protein glycation by decreasing reactive intermediate (methylglyoxal), advanced glycation end products (Nε-(carboxymethyl) lysine), and their receptors (AGEs), and antagonized oxidative stress and nuclear factor-κB activation.

Projecting the beneficial effects of betanin found in the present study to human beings, an individual with a body mass of 80 kg could benefit from the physiological effects of betanin by drinking approximately 90 mL/day of beetroot juice, considering both the betanin concentration (1.19 mg·mL^−1^) in fresh juice and its gastrointestinal stability [31]. It is worth mentioning that beetroot is also a source of other bioactive compounds such as NO_3_^−^, polyphenols, organic acid, and vitamin C [30], which, due to their biological effects, may act synergistically with betanin, making the intake of doses less than 90 mLstill effective.

## 5. Conclusions

The administration of 20 mg·kg^−1^ purified betanin by oral gavage for 20 days was able to reduce oxidative stress through glucose, insulin, and insulin resistance level regulation and reverse hepatic damage through the reduction of AST and ALT levels inWistar rats fed a hyperlipidemic diet (60% kcal as fat) for 60 days. In addition, betanin ingestion increased GPx, CAT, and SOD enzyme activity and reduced hepatic MDA and lipid accumulation in hepatocytes. In the present study, two limitations should be scored and overcome in future studies: the evaluation of a possible modulation of the gene expression by betanin that would increase GPx, CAT, and SOD activities, explaining the increased enzymatic activities observed herein, and an extension of the betanin effect for female animals, to produce precise and reproducible results that could be applicable to both men and women.

These findings reinforce randomized, controlled, and crossover studies concerning dietary betanin supplementation by beetrootcereal bars in individuals presenting risk factors for cardiovascular diseases. Betanin is a bioactive compound which may act as an adjuvant in the treatment and prevention of chronic and degenerative diseases related to oxidative stress in humans.

## Figures and Tables

**Table 1:** Ingredients and nutritional feed composition (100 g).

Ingredients	Standard AIN-93G	High-Fat AIN-93G
Casein (g)	20	20
Starch (g)	52.9	27.95
Soy oil (g)	7	7
Lard (g)	0	25
Sugar (sucrose, g)	10	10
Minerals (mg)	1.52	1.52
Calcium	500	500
Phosphorus	300	300
Magnesium	50	50
Sodium	104	104
Potassium	360	360
Chloride	163	163
Sulfur	30	30
Iron	4	4
Zinc	4	4
Manganese	1	1
Vitamins	5.5	5.5
Nicotinic acid (mg)	1.5	1.5
Pantothenic acid (mg)	1.5	1.5
Pyridoxine (mg)	0.6	0.6
Thiamine (mg)	0.5	0.5
Riboflavin (mg)	0.6	0.6
Folic acid (mg)	0.2	0.2
Biotin (mg)	0.002	0.002
Vitamin B12 (µg)	2.5	2.5
Vitamin K (µg)	90	90
Vitamin E (µg)	400	400
Vitamin A (µg)	120	120
Vitamin D (µg)	3	3
l-Cysteine (g)	0.3	0.3
Colin (g)	0.25	0.25
Cellulose (g)	5	5
Total	100	100
PTN (g)	17.3	17.3
CHO (g)	54.2	29.2
LIP (g)	7	32
SFA (g)	1.1	10.9
MUFA (g)	1.7	12.9
PUFA (g)	3.6	6.4
Cholesterol (g)	0	0.02
Total fiber (g)	5	5
kcal	349	474

kcal—kilocalories; PTN—protein; CHO—carbohydrate; LIP—lipids; SFA—saturated fatty acids; MUFA—monounsaturated fatty acids; PUFA—polyunsaturated fatty acids.

**Table 2:** Plasma biochemical parameters after phase 1 intake of distinct feeds.

	Phase 1 Study	
Parameters	CONT 60	HF 60
Glucose (mg·dL^−1^)	83.3 ± 3.1	120.2 ± 8.0*
Insulin (µUi·mL^−1^)	32.9 ± 2.6	38.5 ± 3.6
HOMA-IR (mmol·L^−1^)	6.7 ± 0.5	11.3 ± 1.2*
TC (mg·dL^−1^)	47.6 ± 2.0	58.1 ± 2.1*
TG (mg·dL^−1^)	27.1 ± 1.9	31.7 ± 4.4*
AST (U·L^−1^)	133.1 ± 8.4	216.2 ± 10.5*
ALT (U·L^−1^)	40.0 ± 3.2	48.4 ± 6.2

Values are expressed as means ± SD. The symbol* denotes difference from CONT 60 at a *p* < 0.05 significance level. CONT 60: control group fed the standard feed AIN-93G; HF 60: high-fat group fed the hyperlipidemicfeed. Animals (*n* = 36) were fed for 60 days. HOMA-IR: insulin resistance assessment by homeostatic model assessment; TC: total cholesterol; TG: triglycerides; AST: aspartate aminotransferase; ALT: alanine aminotransferase.

**Table 3:** Plasma biochemical parameters after phase 2 of feed supplementation.

Phase 2 Study				
Biochemical Parameters	CONT 80	CONT 80+BET	HF 80	HF 80+BET
Glucose (mg·dL^−1^)	124.9 ± 12.3^a^	112.6 ± 16.5^a^	137.2 ± 23.4^b^	106.6 ± 8.3^a^
Insulin (µUi·mL^−1^)	42.7 ± 5.3^a^	36.7 ± 6.9^a^	59.8 ± 4.9^b^	39.4 ± 7.1^a^
HOMA-IR (mmol·L^−1^)	13.2 ± 2.3^a^	10.2 ± 2.1^a^	20.4 ± 4.9^b^	10.9 ± 1.7^a^
TC (mg·dL^−1^)	43.7 ± 7.7^a^	45.7 ± 3.9^a^	69.7 ± 10.8^b^	71.7 ± 5.5^b^
TG (mg·dL^−1^)	20.3 ± 5.1^a^	19.6 ± 3.1^a^	38.8 ± 8.6^b^	28.6 ± 5.9^a^
AST (U·L^−1^)	193.0 ± 74.5^a^	172.0 ± 49.5^a^	265.5 ± 60.3^b^	137.6 ± 27.3^a^
ALT (U·L^−1^)	68.4 ± 2.8^a^	56.1 ± 10.4^b^	78.2 ± 7.3^c^	37.6 ± 7.2^b^

Values are expressed as means ± SD. Different letters on the same line indicate differences between CON T80, CONT 80+BET, HF 80, and HF 80+BET at a *p* < 0.05 significance level. CONT 80: control group fed the standard feed AIN-93M; CONT 80+BET: control group fedthe standard feed plus betanin (20 mg·kg^−1^); HF 80: high-fat group fed the hyperlipidemic feed; HF 80+BET: high-fat group fed the hyperlipidemic feed plus betanin (20 mg·kg^−1^). HOMA-IR: insulin resistance assessment by homeostatic model assessment; TC: total cholesterol; TG: triglycerides; AST: aspartate aminotransferase; ALT: alanine aminotransferase.
